# Effect of postbiotics on the production parameters of rearing goose

**DOI:** 10.1016/j.vas.2025.100541

**Published:** 2025-11-11

**Authors:** Stella Szabó, Mária Kovács-Weber, Tibor István Pap, Ágnes Dolányi, Ferenc Pajor, Ákos Bodnár, Tamás Vajda, Mónika Heincinger, Rubina Tünde Szabó

**Affiliations:** a Institute of Animal Husbandry, Gödöllő Campus, Hungarian University of Agriculture and Life Sciences, H-2100 Gödöllő, Hungary; b Lab-Nyúl Ltd. Malomtó u. 8., H-2100 Gödöllő, Hungary; c Integrál Zrt. Szalay Gyula park 1., H-6900 Kiskunfélegyháza, Hungary

**Keywords:** Postbiotic, Fattened liver, Liver colour, Stiffness

## Abstract

Our aim was to measure the mortality, live body weight, feed conversion, intake and detect the weight and stiffness of the liver, liver colour, breast and thigh weight as an effect of the dry form of Burain® postbiotic supplementation in rearing goose. The goslings were allocated into 5 equal groups: control (without supplementation), group 1 (1 g/kg postbiotic during the starter and grower period), group 2 (2 g/kg postbiotic during the starter and grower period), group 3 (0.2 g/kg postbiotic during the starter and grower period), group 4 (1 g/kg postbiotic during the starter period). Granulated feed and drinking water were provided ad libitum to all rearing goose. Supplementation significantly increased body weight in group 2 (6321.84±664.9 g) on week 8. Group 2 showed a significantly reduced feed conversion ratio (1.51±0.04), while groups 1 and 4 exhibited significantly lower daily feed intake than the control during the starter phase. There were no significant differences among groups in foie gras parameters or in thigh and breast weights. The liver weight and stiffness were found to be significantly positively correlated in each treatment group. The supplementation made a significant difference in the parameters of b* and L* for liver colour, but not for a* values. The impact of postbiotic supplementation in the rearing period is extended to weeks. Further investigations are needed to connect information from rearing to the fattening phase and the gut microbiome in goose.

## Introduction

The production process of fattened goose and fattened liver (foie gras) requires great care to ensure the quality of the final product (Adli et al., 2022; Wojtycza et al., 2025). Housing conditions, the attitude of the caretaker, and the application of the overfeeding are critical for waterfowl species, especially for goose. In addition to housing and handling, feed and feed supplementations are also important factors in the quality of the foie gras (Adli et al., 2022). The elimination or reduction of the use of antibiotics as growth promoters is a focus to avoid health problems of birds (Reuben et al., 2021; Jiang et al., 2024). Antimicrobial resistance, the growing concerns about the harmful effects of industrial-scale farming, and increasing consumer demand for chemical- or antibiotic-free products have made it essential to identify and use safe, natural, and economical alternatives (Reuben et al., 2021; Mansoor et al., 2023; Phupaboon et al., 2024). The family of ‘biotics’ (prebiotics, probiotics, synbiotics, and postbiotics) has a huge potential to improve the biological and production parameters of farm animals and aquaculture (Zamojska et al., 2021; Naveed et al., 2024) including waterfowl (Rashid et al., 2023; Mehmood et al., 2023; Alqahtani et al., 2024; Magnoli et al., 2024). As is known, the microbiota-host symbiosis is essential for the normal development of the immune and nervous systems, for adaptation to environmental influences, and for maintenance of poultry health (El-Ghany, 2020). Studies primarily centered mostly on prebiotics and probiotics, then interest focused on postbiotics in poultry species (Reuben et al., 2021). Probiotics are ‘live microbes that are beneficial for the host’s health,’while postbiotics are ‘intact non-viable microbes or cell fragments, with or without metabolites that provide a health benefit’ (Vinderola et al., 2022). Postbiotics are bioactive compounds synthesized by the general metabolic activity of bacteria (lactic acid bacteria), including during the metabolism of prebiotics. (El-Ghany, 2020; Vinderola et al., 2022). Currently, the most common postbiotics are made from Lactobacillus and Bifidobacterium strains. Postbiotic metabolites can be categorized: short chain fatty acids, exopolysaccharides, enzymes, cell-free supernatants, lysates or other metabolites.

Bacterial lysates are made from Gram-negative and Gram-positive bacterial cells that are broken down and can stimulate the immune system against infections. The positive effects on human lung health (respiratory tract infections in children or pulmonary disease in adults) are observed (Thorakkattu et al., 2022). In poultry production, the application of postbiotics in the feed system as an alternative to antibiotic-containing productivity enhancers has a number of beneficial effects (poultry health, growth performance, meat quality, gut microbiota composition, antioxidant levels and feed conversion). These effects of postbiotics can be attributed to different mechanisms, including promotion of feed digestibility and gut morphology, competitive exclusion and antagonism of pathogens, modification of the gut microbiota, production of antimicrobial compounds, and activation of the immune system.

Monika et al. (2024) showed that postbiotics from *Lactobacillus acidophilus* had relevant and effective impact on growth parameters, health, and gut structure in chickens. Dose of 0.2 % postbiotic supplementation had significantly increased the body weight, the feed conversion ratio (FCR), jejunal antioxidant values and the immune responses. Significant reductions in total plate counts, coliform counts and maximum Lactobacillus counts were observed in all postbiotic-supplemented groups. All postbiotic groups had significant improvement in villus height, width and crypt depth on the 21st and 42nd days of the experimental trial. In the case of the function and viability of the liver. There was a non-significant decrease in the level of serum glutamic oxaloacetic transaminase and serum glutamate pyruvate transaminase enzymes may be expressed less liver damage. El-Ghany et al. (2022) recommended postbiotic compound either in a dry and/or an aqueous form. Combined feed and water treatments with the postbiotic compound reached the highest protection rate (93 %) against *Escherichia coli* and showed no signs of liver congestion, necrosis, or enteritis. The highest significant average body weights were recorded in chickens treated with combined postbiotic treatments in feed and water (1360.7 g), followed by feed (1245.9 g) and water (1196.5 g). In groups treated with the postbiotic in feed and combined feed and water, liver showed normal hepatic parenchyma, while postbiotic treatment in water revealed focal portal hepatitis and moderate enteritis.

Different probiotics have been used to improve goose production. In the work of Yu et al. (2021), probiotics promoted feed intake and growth, improved antioxidative capacity, intestinal morphology, and intestinal microbial composition in Yangzhou geese. Among similar results, probiotic compounds presented higher immunoglobulin A, superoxide dismutase, catalase, total antioxidant capacity and glutathione peroxidase levels (Chen et al., 2023). Probiotic supplement in water improved dressing and the liver weight, decreased the triglycerides, total cholesterol, LDLcholesterol, and aspartate aminotransferase level in native Turkish geese (Ölmez et al., 2022).

In preliminary postbiotic studies, doses commonly ranged from 0.05 to 0.6 % and were utilized mostly in broiler chickens or layers (Gingerich et al., 2021; Pearlin, 2021; Danladi et al., 2022; El-Ghany et al., 2022; Saeed et al., 2023; Al-Salhi et al., 2024). In our previous study, 4 g Burain® postbiotic/ 1 kg feed resulted higher live weight and weight gain in the mixed sex chicken group, however the feed conversion was the same in control and postbiotic groups (Szabó et al., 2024). The formulation of the product is known in humans (Framelim, Kardamom Pharma Kft, Tiszabura, Hungary); for its use in geese, we relied on literature and our preliminary results in chicken (Szabó et al., 2024). These low supplementation dosages focused on cost-effectiveness in goose rearing.

To the best of our knowledge, there is no previous postbiotic supplementation study in geese, where evaluated the effect of postbiotics (0.2, 1.0 and 2.0 g/kg) on goose growth performance and foie gras quality; therefore, the aim of this study was to evaluate the efficacy of postbiotic lysates from *Lactobacillus reuteri*, L. *acidophilus* and *Bifidobacterium longum* on natural indicators such as liver weight, foie gras colour, stiffness, live body weight and feed intake. We hypothesized that the inclusion of postbiotic lysates in the diet of geese improves the growth performance, and its positive effect can be evident in liver parameters without supplementation during the force-feeding period. By this information, we could get a view of the effect of the postbiotic administered during the rearing period (before the overfeed) as reflected in the final product, in particular on the weight and quality of the liver, if no supplementation was added during the overfeeding phase.

## Material and methods

### Ethic statement

The experimental protocol was authorized by the Workplace Animal Welfare Committee of Hungarian University of Agriculture and Life Sciences (MATE-MKK-2020/22) because this work was not covered by 40/2013 (II. 14) government decree on animal experiments.

### Birds and experimental design

A total of 600 day-old goslings (INTEGRAL MB 09 hybrid, Kisbéri Lúdtenyésztő Kft., Hungary) of mixed sex were obtained from a local hatchery, AB OVO Kft. (Hungary). The goslings were allocated into 5 equal groups (1–4, control) consisting of 120 birds, each assigned into 6 equal replicates (20 each). Vaccination was done against *Riemerella anatipestifer* and *Salmonella enterica* subcutaneously at 10 days old. All the birds were kept in a deep litter system (chopped straw), 20 animals were kept in one unit (2 × 3.15 m). For the first 4 days, the number of light hours was 24 h, then it decreased gradually, by 1 h per day, to 17 h until the end of the period.

Birds were fed on starter and grower diets at ages 1–21 and 22–56 days, respectively. The composition of the commercial balanced diets is presented in Table 1.

**Table 1:** Composition of diets.

Starter (days 1–21)		Grower (days 22–56)	
Moisture content %	14.00	Moisture content %	14.00
Crude protein %	20.00	Crude protein %	18.00
Crude fat %	3.00	Crude fat %	3.70
Crude fibre %	4.30	Crude fibre %	5.40
Crude ash %	5.62	Crude ash %	5.00
Lysin %	1.06	Lysin %	0.97
Metionine %	0.50	Metionine %	0.46
Calcium %	1.00	Calcium %	0.85
Total phosphorus %	0.73	Total phosphorus %	0.58
Sodium %	0.15	Sodium %	0.15
Vitamin A, NE/kg	10,000.00	Vitamin A, NE/kg	10,000.00
Vitamin D3, NE/kg	3750.00	Vitamin D3, NE/kg	3750.00
Hy-D3, NE/kg	0.00	Hy-D3, NE/kg	0.00
Vitamin E, NE/kg	50.00	Vitamin E, NE/kg	50.00

Table 1: Composition of diets

Granulated feed and drinking water were provided ad libitum. The dry form of Burain® (Kardamom Pharma Kft, Tiszabura, Hungary; EU Reg. No. 10 1 00,307) was applied as feed supplementation in four treatment groups. Group 1–3 were supplemented with Burain® during the whole rearing period (56 days); supplementation of group 4 was only in the starter feed (21 days) (Table 2).

**Table 2:** Dosage of the postbiotic supplementation in control and experimental groups.

Group	Starter period	Grower period
1	1 g/kg	1 g/kg
2	2 g/kg	2 g/kg
3	0.2 g/kg	0.2 g/kg
4	1 g/kg	-
control	-	-

Starter period: week 1–3, Grower period: week 4–8.

Table 2: Dosage of the postbiotic supplementation in control and experimental groups

The Burain® postbiotic (1 kg) contained 500 g lysate: 300 g Lactobacillus reuteri SG L 01, 100 g L. *acidophilus* SG L 11, and 100 g Bifidobacterium longum SGB 05. The substrate was 250 g wholegrain oatmeal and 250 g oat bran to the lysate. After the growing period, an overfeeding period (13 days) was taken without any supplementation. The starting amount of feed (100 g/bird/meal) was increased to 400 g/day. Each group of animals was given two meals per day, and the number of meals was increased to four. The diet included grain maize (28 %), maize semolina (70 %), and premix (2 %). After the overfeeding period, the geese were slaughtered at a high-capacity slaughterhouse (Integrál Zrt., Kiskunfélegyháza, Hungary). The distance to the slaughterhouse was 193 km, the transportation was taken before 6 am.

### Measured parameters

Mortality and feed consumption (intake) values were recorded daily. The feeders had constantly hung on Wi-Fi-connected scales (Spirocco, Budapest, Hungary), and the weight of the feed was measured at the set time. The feed conversion ratio was calculated for the starter and for the grower period. Live body weight was weekly assessed in all groups through the 8-week experimental period and was recorded just before the slaughtering (Veit Electronics, BAT1, Moravany, Czech Republic). During the overfeed period, the live body weight was not recorded. The thigh, breast (right and left), and foie gras (fattened liver) were weighed after 24 h of cooling.

The CIELAB system was utilized to evaluate liver colour parameters (lightness, L*; redness, a*; yellowness, b*). Measurements were taken by Minolta R-400 Chromameter with an 8 mm head (Konica Minolta Sensing, Inc., Osaka, Japan). The colour apparatus was calibrated against a white calibration plate before each reading.

The stiffness measurements were performed using a TA.XT Plus texture analyzer (Stable Micro Systems, Godalming, Surrey, UK) equipped with a spherical probe. The stiffness was calculated based on the force per unit time (g) diagram using Exponent Connect software. The measurements were carried out on the right cranial end of the liver, on the ventral surface of the abdominal part. The spherical probe was inserted at a depth of 2 cm vertically into the specimen with three technical replicates.

### Statistical analyses

Statistical analysis was processed using the R software package (R Core Team R, 2013). Shapiro–Wilk’s test was performed to test the normality distribution. The groups were compared with one-way ANOVA tests followed by Tukey post hoc tests. Correlation analysis was performed using Pearson’s correlation with pairwise comparisons to determine simple correlation coefficients. Significant differences between treatment results were considered significant at level p ≤ 0.05.

## Results

### Body weight

There were no significant differences in mortality. Table 3 presents the effect of postbiotic supplementation on live body weight during the rearing period.

**Table 3:** Results of postbiotic treatments on live body weight (g).

	Groups					p-value
	Control	Group 1	Group 2	Group 3	Group 4	
Day 1	98.03± 10.30a	96.69± 12.59a	95.82± 12.35a	96.95± 11.87a	98.46± 10.33a	0.398
Week 1	293.25± 63.36a	301.77± 60.69a	296.15± 48.74a	291.99± 66.33a	295.10± 55.37a	0.789
Week 2	716.01±150.89a	763.10±104.20a	732.06±123.06a	738.01± 121.45a	734.35± 131.52a	0.122
Week 3	1458.05±198.64a	1503.21±164.41a	1458.58±178.55a	1468.33±185.56a	1471.46±170.66a	0.377
Week 4	2352.91±260.13a	2389.36±260.73a	2350.30±236.93a	2367.85±274.20a	2413.93±258.01a	0.364
Week 5	3178.95±326.54a	3216.50±309.77a	3193.92±284.59a	3219.21±346.35a	3291.89±323.01a	0.117
Week 6	3785.31±384.53a	3796.52±375.12a	3804.04±334.05a	3809.87±424.60a	3905.85±416.02a	0.186
Week 7	4263.67±310.37a	4345.15±423.68a	4293.07±392.09a	4368.58±488.21a	4397.63±413.25a	0.123
Week 8	4489.12±337.96a	4588.29±398.70ab	4628.57±327.98b	4464.90±389.77a	4542.50±366.62ab	0.004
Before slaughter	6238.46± 590.52ab	5833.79±644.9a	6321.84±664.9b	6158.71± 720.11ab	6029.28± 777.05ab	0.040

a,b: different superscript letters show significant differences (*p* ≤ 0.05) between groups in the same week by Tukey test.

Table 3: Results of postbiotic treatments on live body weight (g)

**Fig. 1:**
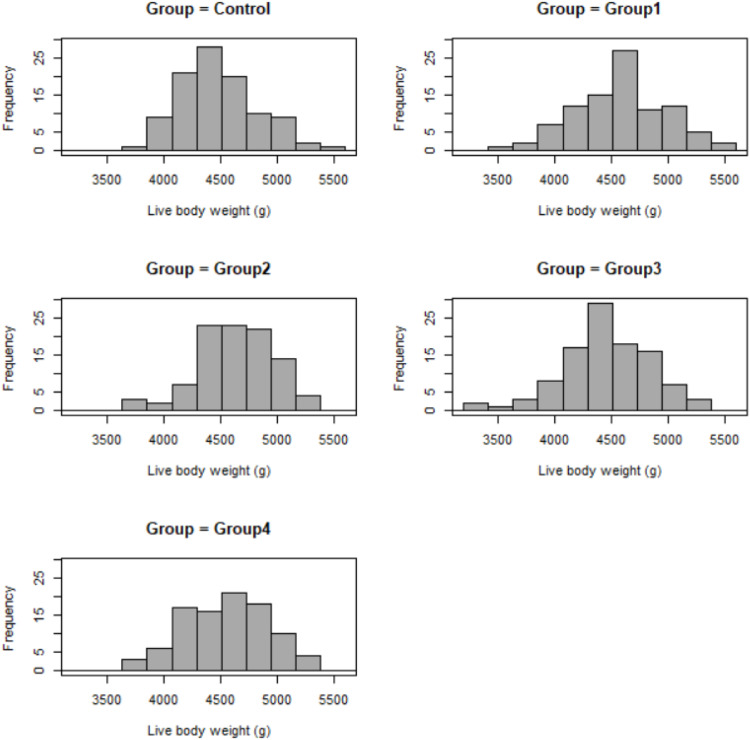
Distribution of live body weight in different groups. y axis shows the number of liver weight in each group; control – no supplementation, *n* = 101; Group1 – 1 g/kg postbiotic supplementation, *n* = 93; Group2 – 2 g/kg postbiotic supplementation, *n* = 98; Group3 – 0.2 g/kg postbiotic supplementation, *n* = 103; Group4 – 1 g/kg postbiotic supplementation, *n* = 94.

The supplementation had no effect on body weight results from week 1 to week 8. Group 2 had significantly increased body weight compared to the control (*p* = 0.005) and group 3 (*p* = 0.02) on week 8 (Table 3). The control, group 1 and 3 showed similar frequencies of different live body weight values, while group 2 represented higher values (above 4500 g) with high frequency (Fig. 1). Group 2 had the highest live body weight before the slaughtering and significantly differed from group 1.

Fig. 1: Distribution of live body weight in different groups

### Feed conversion, feed intake

During the starter phase, the feed conversion ratio of group 2 was significantly lower than that of the control (*p* = 0.01) and group 4 (*p* = 0.008); however, there were no differences during the grower phase or the total rearing period (Table 4).

**Table 4:** Results of postbiotic treatments on feed conversion ratio in different group and period.

FCR (kg/kg body weight)	Group					p-value
Phase	Group 1	Group 2	Group 3	Group 4	control	
Starter	1.57±0.03ab	1.51±0.04a	1.55±0.02ab	1.61±0.05b	1.60± 0.05b	0.005
Grower	3.97± 0.37	3.99± 0.49	4.00± 0.32	4.06± 0.27	4.12± 0.23	0.95
Rearing (starter+grower)	3.17± 0.18a	3.19±0.28a	3.22±0.18a	3.28± 0.17a	3.30±0.15a	0.744

a,b: different superscript letters show significant differences (*p* ≤ 0.05) between groups in the same phase by Tukey test.

Table 4: Results of postbiotic treatments on the feed conversion ratio in different groups and periods

Oppositely, the average daily feed intake differed in the grower period but not during the starter phase. Group 1 and 4 had significantly lower daily feed intake values compared to the control (*p* = 0.001), and group 1 significantly differed from group 2 (*p* ≤ 0.001) and 3 (*p* = 0.04) (Table 5).

**Table 5:** Results of postbiotic treatments on daily feed intake in grower period.

Grower period	Groups					p-value
	Control	Group 1	Group 2	Group 3	Group 4	
Daily feed intake, g	5856.92±121.0a	5285.83±105.3b	5745.52±115.1ac	5842.62±104.6a	5534.36±103.3bc	< 0.0001

a,b: different superscript letters show significant differences (*p* ≤ 0.05) between groups in the grower phase by Tukey test.

Table 5: Results of postbiotic treatments on daily feed intake in the grower period

### Liver parameters

**Fig. 2:**
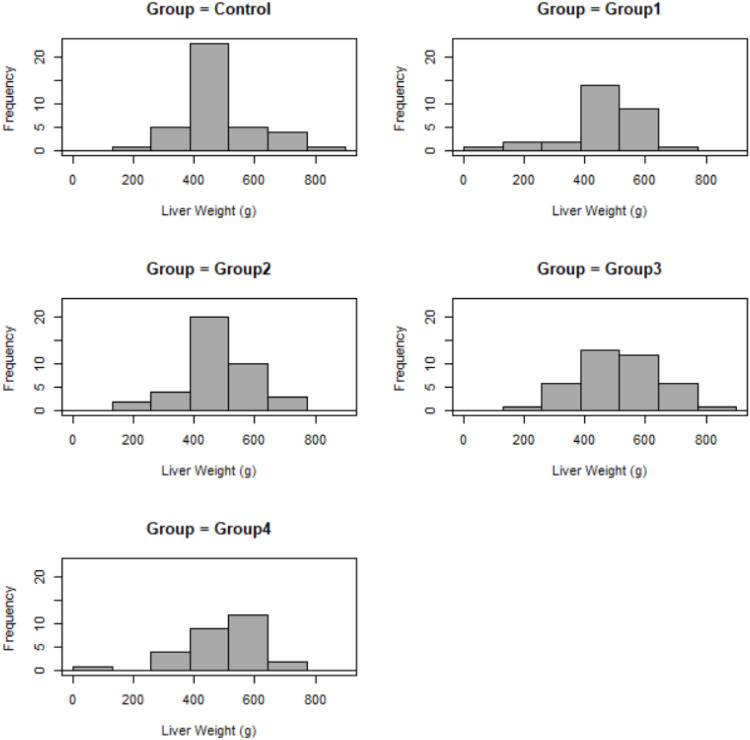
Distribution of liver weight in different groups. y axis shows the number of liver weight in each group; control – no supplementation, *n* = 38; Group1 – 1 g/kg postbiotic supplementation, *n* = 28; Group2 – 2 g/kg postbiotic supplementation, *n* = 38; Group3 – 0.2 g/kg postbiotic supplementation, *n* = 38; Group4 – 1 g/kg postbiotic supplementation, *n* = 28.

The weight and stiffness of foie gras and the weight of the thigh and breast showed no significant differences between groups. In each treatment group, a strongly positive significant correlation was detected between liver weight and stiffness (*p* ≤ 0.001, *r* = 0.65–0.77). The liver weight frequency distribution was similar in the control and in group 2 (Fig. 2).

Fig. 2: Distribution of liver weight in different groups

In the case of liver colour, the supplementation resulted in significant differences in parameters of b* (*p* < 0.0001) and L* (*p* < 0.0001), but a* values did not differ between groups. Group 1 had the highest b* value, significantly higher than the control (*p* = 0.001) and group 2 (*p* < 0.0001). The highest supplementation (group 2) resulted in the lowest b* value, differed from all other supplemented groups (Table 6). Group 2 had the darkest red liver and had significantly lower L* value compared to the control and other supplemented groups. A negative significant correlation was determined between L* and a* values in group 1 and 4 (*p* ≤ 0.0001, *r*=−0.8984; −0.7075). Between L* and b*, results showed a positive significant correlation was found in group 2 (*p* ≤ 0.0001, *r* = 0.8236).

**Table 6:** Results of postbiotic treatments on liver, thigh and breast weight and liver colour in different groups.

Parameters	Groups					p-value
	Control	Group 1	Group 2	Group 3	Group 4	
L*	63.64±4.72c	72.55±5.99a	57.19±3.02b	71.76±4.58a	70.14±6.97a	< 0.001
a*	5.00±1.43a	5.72±1.29a	4.89±1.86a	5.13±1.28a	5.34±1.65a	0.322
b*	19.93±2.77bc	22.55±2.32a	18.21±2.61c	21.16± 2.21ab	21.16± 2.81ab	<0.001
Liver weight (g)	485.71±126.58a	466.51±126.37a	477.02±116.87a	517.89±131.51a	484.92±130.04a	0.502
Stiffness (g)	458.55±201.11a	479.54±260.53a	499.48±226.88a	526.82±249.01a	483.54±218.63a	0.762
Thigh (left)	420.89±54.54a	399.62±55.22a	432.23±46.35a	432.64±58.58a	424.17±54.79a	0.100
Thigh (right)	417.43±54.85a	406.24±60.36a	438.61±54.94a	432.74±49.46a	428.35±62.50a	0.136
Breast (left)	276.28±36.62a	262.96±32.72a	279.25±39.68a	265.38±35.70a	269.67±43.98a	0.310
Breast (right)	271.97±34.80a	257.00±42.50a	282.10±37.83a	268.23±37.68a	274.21±43.24a	0.124

a,b: different superscript letters show significant differences (*p* ≤ 0.05) between groups in the same parameter by Tukey test.

Table 6: Results of postbiotic treatments on liver, thigh, and breast weight and liver in different groups

## Discussion

The poultry sector is one of the leading growing animal husbandry industries contributing to antibiotic usage. Antibiotics have several benefits in the prevention and treatment of disease, can improve the health and animal welfare of poultry, or improve the growth performance parameters. Antibiotics in the poultry field have focused on concerns about residues in meat and egg products and the development of antibiotic-resistant bacterial populations (bacteria or genes), and because of it, these can lead to adverse effects on the health of poultry and humans. (El-Ghany, 2020; Muhammad et al., 2020; Barros et al., 2023). Because of these issues, many studies aimed to test replacements for antibiotics with profitable effects on poultry production and quality. The family of ‘biotics’ (prebiotics, probiotics, synbiotics, and postbiotics) has been extensively investigated and has great potential in replacing antibiotics. Until now, postbiotic research mostly focused on chickens, and there is little knowledge of the effects of postbiotics in goose.

### Body weight

In our study, the highest significant average body weight was recorded in goose treated with 2 g/kg postbiotic supplementation on week 8, before starting the overfeeding period. Other experimental groups did not significantly differ but were more favorable in live body weight parameters than the control group. These results indicated that the postbiotic improved live body weight results. The same trend was observed in chickens with Lactobacilli-based postbiotic supplementation. In the work of El-Ghany et al. (2022), by the end of the experiment, all treated groups significantly differed from control results. The combination of postbiotic treatments in feed and water resulted in the highest body weight values. Jansseune et al. (2024) presented that Lactobacillus-based probiotic or postbiotic supplementation can react similarly to higher body weight, resulting in a standard or challenge diet compared to the non-supplementation group. In broiler goose production, probiotic (*Clostridium butyricum* and *Bacillus subtilis*) treatment improved the production performance of geese in the middle and the end of the rearing (day 28 and 70) (Yu et al., 2021). Li et al. (2023) and Li et al. (2025) also confirmed that probiotic (*Bacillus subtilis*) supplementation significantly increased the final body weight of geese. Because of the lack of information about the postbiotic effect on goose, it is suggested to test Burain® postbiotic supplementation in broiler goose rearing, where maximizing body weight is the goal.

### Feed conversion, feed intake

In our study, 2 g/kg postbiotic supplementation significantly reduced the feed conversion ratio and resulted in the highest body weight at the end of the rearing. Similarly, postbiotic supplementation resulted in better body weight values in chickens (El-Ghany et al., 2022). Conversely, in the work of Yu et al. (2021), Li et al. (2023), and Jansseune et al. (2024), the feed conversion ratio did not differ by probiotic or postbiotic treatments. In the work of Li et al. (2025), FCR showed significantly higher values by probiotic supplementation in geese. Contrary, Chen et al. (2025) and Sun et al. (2025) represented improved FCR values with different probiotic supplementations. The gastrointestinal tract microbiota population structure is on of the key factors to the efficiency in converting food into muscle mass (Stanley et al., 2012; Yue et al., 2025). The cecal microbial community displayed higher diversity in high performing chickens. Numerous bacteria can serve as target populations that might be altered by various biotics to enhance animal growth performance (Stanley et al., 2012). The probiotic supplementation improved the feed consumption (intake) in the study of Ölmez et al. (2022), likely in our group 1 (1 g/kg in the starter and grower periods) and 4 (1 g/kg in the starter period), but only in the grower period. By contrast, feed consumption values of mule ducks rose in probiotic supplementation groups.

### Liver parameters

Wen et al. (2022) elaborated that postbiotic *Lactobacillus* can decrease live body weight and fattened liver weight, which can be related to the transport of nascent fat from the liver to abdominal fat. In a 90-day-long experiment, Xu et al. (2022) introduced the effect of probiotic-rich brewers’ spent grain on goose performance. The 4 % supplementation did not result in significant differences during the rearing or during the overfeeding periods compared to the control groups, but when the bird got the supplementation during the rearing and overfeeding stages, the liver weight significantly increased. However, in mule duck probiotic treatment (*Lactobacillus salivarius*) did not result in any changes in liver weight values during the overfeeding period (Even et al., 2018). In our study, the Burain® postbiotic supplementation did not inhibit the fat metabolism but did not result in significant differences in liver weight.

The colour of any product is always important for the consumers. In our experiment, group 1 had significantly lighter (L*=72.55) and significantly yellower (b*=22.55) fattened liver. These results are favourable because the yellower (clay yellow) and the lighter fattened liver is preferred by consumers (Fernandez et al., 2019). In the case of red colour, the pink cream, light red, is preferred in the market (Gerzilov et al., 2020). In this study, the supplementation did not affect the red colour significantly, the results were between 4.89 and 5.72. There was a negative significant correlation between L* and a* values in group 1 and 4, therefore it may be concluded that the use of the Burain® postbiotic supplementation can be more reasonable in the grower period. There is limited information about goose liver colour modification by probiotics or postbiotics. In previous probiotic supplementation study, there was no significant differences in breast colour (Chen et al., 2025). Postbiotics can modulate lipid absorption and metabolism, can increase energy expenditure in liver (Nazarinejad et al., 2025). The redness changes of liver can be the consequence of lipid oxidation of fatty acids, because postbiotics promote the synthesis of short-chain fatty acids (SCFAs) (An et al., 2025). Lipid metabolism modulation may increase or decrease lightness, depending on fat deposition, distribution of foie gras. Oxidative stress can reduce L* and increase b*, lead to discoloration. Postbiotics may have beneficial impact on lipid accumulation and composition, more unsaturated fats can be favourable to rise b* values (Wei et al., 2020). The fatty liver syndrome can result more stiffness, more rupture and damage in the structure of the liver (Bhatt et al., 2021). This syndrome did not develop; the stiffness of the supplemented groups did not differ from control values in our study. Chen et al. (2025) came to the same observation in the case *Clostridium butyricum* and *Bacillus subtilis* supplementation in geese. The Burain® postbiotic supplementation did not affect the weight of breast and thigh. A similar finding was reported by Liu et al. (2025) who demonstrated no changes in breast muscle weight percentage and leg weight percentage by probiotic *Bacillus subtilis* and Bacteriophage supplementation. Chen et al. (2025) described the same trend in leg muscle yield, but significant increase was found in breast muscle yield in *Clostridium butyricum* and *Bacillus subtilis* probiotic supplementation groups.

In conclusion, sustainable poultry production with postbiotics is feasible and may be an option to maintain the optimal gut health in goose to maximize production. In goose fattened liver production, the live body weight before the overfeeding phase is crucial; our 2 g/kg supplementation could result in the best result. The supplementation in the rearing period and no supplementation in the overfeeding period affected the liver colour parameters, the impact of postbiotics extended to weeks. 1 g/kg postbiotic achieved the most favourable fattened liver colour. There is a need for further research to collect information about the effect of postbiotics during the fattening phase and the gut microbiome.

## Funding

This study was funded by GINOP_PLUSZ-2.1.1-21-2022-00126, Development related to a breeding and farming technology of premium, antibiotics free foie-gras production that meets the high-level expectation of animal welfare and National Talent Program, Grant number: NTP-SZKOLL-25-0005.

## CRediT authorship contribution statement

**Stella Szabó:** Writing – original draft, Visualization. **Mária Kovács-Weber:** Project administration, Funding acquisition, Conceptualization. **Tibor István Pap:** Investigation. **Ágnes Dolányi:** Resources. **Ferenc Pajor:** Writing – review & editing, Supervision. **Ákos Bodnár:** Methodology, Investigation. **Tamás Vajda:** Resources. **Mónika Heincinger:** Funding acquisition, Data curation. **Rubina Tünde Szabó:** Writing – original draft, Formal analysis.

## Declaration of competing interest

The authors declare the following financial interests/personal relationships which may be considered as potential competing interests Rubina Tunde Szabo reports financial support and administrative support were provided by National Talent Program NTP-SZKOLL-25–0005. Maria Kovacs-Weber reports financial support and equipment, drugs, or supplies were provided by GINOP_PLUSZ-2.1.1–21–2022–00,126. If there are other authors, they declare that they have no known competing financial interests or personal relationships that could have appeared to influence the work reported in this paper.
